# Oscillatoxin I: A New Aplysiatoxin Derivative, from a Marine Cyanobacterium

**DOI:** 10.3390/toxins11060366

**Published:** 2019-06-21

**Authors:** Hiroshi Nagai, Shingo Sato, Kaori Iida, Kazutaka Hayashi, Mioko Kawaguchi, Hajime Uchida, Masayuki Satake

**Affiliations:** 1 Department of Ocean Sciences, Tokyo University of Marine Science and Technology, Tokyo 108-8477, Japan; ss.e.p.akuroi.do45@gmail.com (S.S.); kaori.id@gmail.com (K.I.); kazu.gt.0308@gmail.com (K.H.); ballet305.fouette@gmail.com (M.K.); 2 National Research Institute of Fisheries Science, Japan Fisheries Research and Education Agency, Yokohama, Kanagawa 236-8648, Japan; huchida@affrc.go.jp; 3 Department of Chemistry, The University of Tokyo, Tokyo 113-0033, Japan; msatake@chem.s.u-tokyo.ac.jp

**Keywords:** aplysiatoxin, cyanobacteria, *Moorea producens*, cytotoxicity, biosynthesis

## Abstract

Cyanobacteria have been shown to produce a number of bioactive compounds, including toxins. Some bioactive compounds obtained from a marine cyanobacterium *Moorea producens* (formerly *Lyngbya majuscula*) have been recognized as drug leads; one of these compounds is aplysiatoxin. We have isolated various aplysiatoxin derivatives from a *M. producens* sample obtained from the Okinawan coastal area. The frozen sample was extracted with organic solvents. The ethyl acetate layer was obtained from the crude extracts via liquid–liquid partitioning, then separated by HPLC using a reversed-phase column. Finally, 1.1 mg of the compound was isolated. The chemical structure of the isolated compound was elucidated with spectroscopic methods, using HR-MS and 1D and 2D NMR techniques, and was revealed to be oscillatoxin I, a new member of the aplysiatoxin family. Oscillatoxin I showed cytotoxicity against the L1210 mouse lymphoma cell line and diatom growth-inhibition activity against the marine diatom *Nitzschia amabilis*.

## 1. Introduction

**Figure 1:**
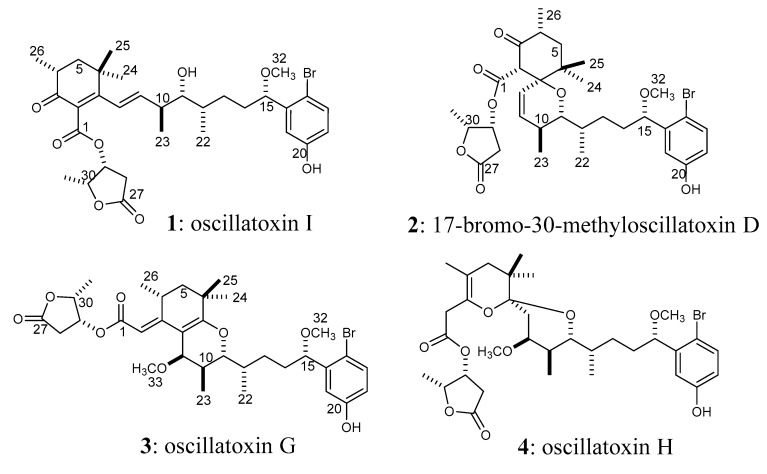
Structures of oscillatoxins obtained from the Okinawan *Moorea producens*.

The marine cyanobacterium *Lyngbya majuscula* is known to produce aplysiatoxins [1,2] and lyngbyatoxins [3,4], which cause severe contact dermatitis [1,3]. Contact dermatitis due to *L. majuscula* has been reported in tropical and subtropical waters, especially in the Pacific region [5,6]. Aplysiatoxin and lyngbyatoxin have also caused food poisoning via the ingestion of the red alga *Gracilaria coronopifolia* [7] and the green turtle *Chelonia mydas* [8], respectively. Recently, blooms of *L. majuscula* have been increasing worldwide, threatening human health and eco-systems; this increase is presumably due to climate change [9]. In Japan, several blooms of *L. majuscula* have also been recorded. During the summer of 2010, a mass occurrence of *Moorea producens* (formerly *L. majuscula*) was observed in the Okinawa Prefecture and lasted for almost one month. A cyanobacterial sample collected at this site during the 2010 mass occurrence was used in this study. In a recent study, we identified 15 aplysiatoxin derivatives in this Okinawan cyanobacteria sample [10]. Aplysiatoxins have been shown to act as protein kinase C (PKC) activators and potent tumor promoting compounds [11,12,13,14,15]; their toxicity results from their high PKC activation potency [11,12,13,14,15]. The compound bryostatin-1, which is obtained from the marine bryozoan *Bugula neritina* [16], is also a potent PKC activator [17]. However, bryostatin-1 has low tumor-promoting activity and potent anti-cancer activity [17,18]. Therefore, bryostatin-1 has been recognized as a lead compound against various cancers [18]. The regulation of PKC activity has been shown to be a valuable therapeutic strategy for producing anti-cancer drugs [19,20]. For example, simplified analogues of aplysiatoxin have been shown to exhibit anti-cancer activity [21,22]. Thus, aplysiatoxins are attractive compounds due to their unique activities. So far, many aplysiatoxins have been isolated and reported from marine cyanobacteria [23,24,25,26,27,28,29]. We have also continued to isolate aplysiatoxin-related compounds from the Okinawan *M. producens* sample. In this paper, the isolation, structural elucidation, and bioactivities of a new aplysiatoxin derivative, oscillatoxin I (**1**, Figure 1), are discussed.

## 2. Results

The frozen sample of the marine cyanobacterium *M. producens* was extracted using MeOH. The obtained methanol extract was subjected to successive separation steps, and finally purified by HPLC using an octadecyl C18 column to yield compound **1** (1.1 mg).

### 2.1. Structural Elucidation of Compound ***1***

**Figure 2:**
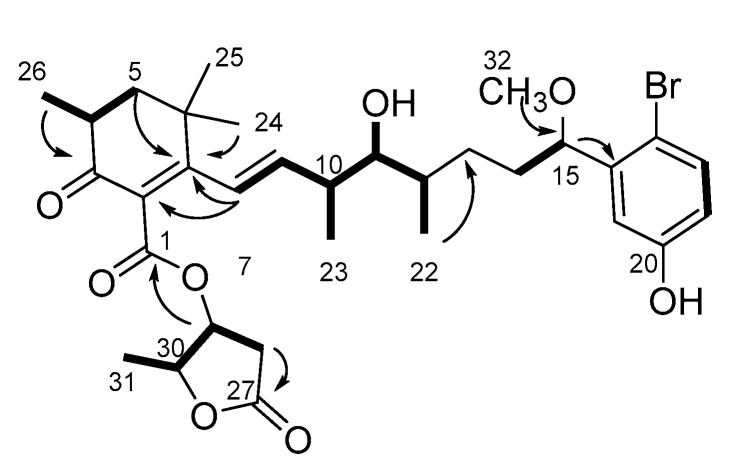
NMR interpretation of oscillatoxin I. Bold line: COSY correlation; Arrow: HMBC correlations.

Compound **1** was isolated as a colorless solid ([α]_D_^17^ +19 (c 0.01, MeOH)). The ^1^H NMR spectrum (Figure S1) showed that compound **1** was an aplysiatoxin-related compound. The molecular formula of compound **1** was deduced to be C_32_H_43_BrO_8_ ([M−H]^−^ 635.2174 and 637.2173, calcd. 635.2220 and 637.2200) (Figure S2), suggesting that compound **1** contained a bromophenol side chain similar to aplysiatoxin. The UV maxima at 228 nm (ε 15,880) and 275 nm (ε 8,190) suggested that compound **1** contained not only a bromophenol chromophore, but also a conjugated system. The HSQC spectra (Figure S3) of compound **1** showed seven methyls (two singlets, four doublets, and a methoxy), four methylenes, three methines bonded to methyls in the aliphatic region, four oxygenated methines, two olefinic methines, and three aromatic protons in the bromophenol side chain and nine quaternary (one aliphatic, two olefinic, three aromatic in the bromophenol, two esters, and one ketone) carbons (Table 1). The carbon signal at δ_C_ 197.4 and an HMBC (Figure S4) correlation from Me-26 (δ_H_ 1.08) to the ketone suggested that compound **1** was related to 17-bromo-30-methyloscillatoxin D (**2**) [10,23]. Analogously to 17-bromo-30-methyloscillatoxin D (**2**), connectivity was observed between the H-4 (Me-26) and H_2_-5 protons in the ^1^H–^1^H COSY spectrum (Figure 2, Figure S5), although the methine singlet of H-2 in 17-bromo-30-methyloscillatoxin D was not observed. HMBC correlations were observed between Me-26 and C-3, and between Me-24 and Me-25 and a quaternary carbon (C-7). The carbon chemical shifts of C-2 at δ_C_ 130.0 and C-7 at δ_C_ 164.0 suggested that these quaternary carbons were olefinic carbons in a conjugated system. These observations revealed that compound **1** contained a cyclohexenone structure. A partial structure from H-8 to H-12 was assigned by analysis the ^1^H–^1^H COSY spectrum. The proton chemical shifts of H-8 and H-9 suggested that these peaks corresponded to olefinic protons; these peaks were shifted downfield from δ_H_ 5.78 and δ_H_ 5.52 in 17-bromo-30-methyloscillatoxin D (**2**) [10] to δ_H_ 6.19 and δ_H_ 6.07 in compound **1**, respectively. In addition to this, HMBC correlations between H-8 and C-2/C-7 were observed. This indicated that H-8 and H-9 also belonged to the conjugated system. The hydroxy proton observed at δ_H_ 3.50 was coupled to H-11, apparently indicating that the hydroxy group was located on C-11. The position of the hydroxy group and the unsaturation number (11) indicated the absence of a six-membered ether ring in compound **1**. The methoxy (Me-32) proton at δ_H_ 3.22 was confirmed to be bonded to C-15 via the HMBC spectrum. The existence of a γ-lactone was deduced from the unsaturation number and the proton chemical shift of H-30 at δ_H_ 4.84, which was close to that of the corresponding peak in 17-bromo-30-methyloscillatoxin D (δ_H_ 4.81) [10]. The HMBC correlation between H-29 at δ_H_ 5.54 and C-1 (δ_C_ 166.6) confirmed the existence of an ester linkage between C-1 and C-29. The proton coupling constants of 4.8 Hz for H-4/H-5b and 13.7 Hz for H-4/H-5a rationalized the equatorial orientation of Me-26 on C-4. The large proton coupling constant (16 Hz) indicated an *E* configuration of Δ^8^. The proton coupling constants of H-11 (11.3 and 1.2 Hz) were similar to those of H-11 in aplysiatoxins that have six-membered ether rings, which suggested that the conformations of H-10-H-11 and H-11-H-12 in compound **1** were likely anti and gauche, respectively, like in aplysiatoxin and its derivatives. The structure of compound **1** is shown in Figure 1. Previously, we reported compounds **3** and **4** as oscillatoxin E and F, respectively [10]. However, the same nomenclature (oscillatoxin E and F) was applied to different aplysiatoxin derivatives by Tang et al. at almost the same time [29]. To avoid confusion, we have renamed compounds **3** and **4** oscillatoxin G and H, respectively. Furthermore, compound **1** was designated as oscillatoxin I.

### 2.2. Biological Activity of Oscillatoxin I *(**1**)*

Oscillatoxin I (**1**) showed 100% inhibition activities in both cytotoxicity and diatom growth inhibition tests at a concentration of 10 µg/mL. The IC_50_ values of oscillatoxin I (**1**) in the cytotoxicity test and diatom growth inhibition test were 4.6 µg/mL and 1.2 µg/mL, respectively.

## 3. Discussion

**Figure 3:**
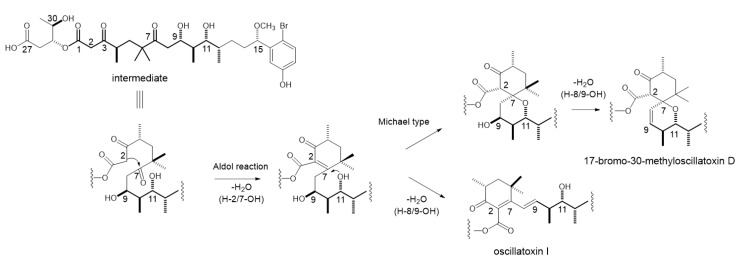
Proposed biosynthetic pathway of 17-bromo-30-methyloscillatoxin D and oscillatoxin I.

Oscillatoxin I (**1**) is an aplysiatoxin analog that lacks a six-membered ether ring in the molecule. The characteristic bicyclo ring systems of aplysiatoxin and its known derivatives have been proposed to be biosynthesized from a common intermediate [10]. The formation of the ring system of 17-bromo-30-methylosicllatoxin D (**2**) is preceded by an intramolecular aldol reaction between C-2 and C-7 and the dehydration of H-2/7-OH to generate a conjugated ketone, followed by an intramolecular Michael-type addition of 11-OH to C-7 (Figure 3). The presence of a cyclohexenone ring in oscillatoxin I (**1**) suggests that the biosynthesis of oscillatoxin I branches from a biosynthetic intermediate of 17-bromo-30-methyloscillatoxin D (**2**). The biosynthesis of oscillatoxin I is presumed to occur as follows: the aldol reaction of C-2/C-7 followed by the dehydration of H-2/7-OH and H-8/9-OH leads to the biosynthesis of oscillatoxin I. The *E* configuration of Δ^8^ prevents the Michael-type addition of 11-OH to C-7. Therefore, the isolation of oscillatoxin I (**1**) reinforced our proposal regarding the biosynthesis of aplysiatoxin derivatives [10].

Osillatoxin I (**1**) showed moderate toxicity in the cytotoxicity test (IC_50_; 4.6 µg/mL) and diatom growth inhibition test (IC_50_; 1.2 µg/mL). We also studied the toxicities of aplysiatoxin and its fourteen derivatives, and found they had mild activities in these bioactivity tests [10]. Oscillatoxin I showed one of the most potent toxicities among aplysiatoxin and its analogues in the cytotoxicity and diatom growth inhibition tests.

## 4. Materials and Methods

### 4.1. General Procedure

HPLC was performed using a Hitachi Chromaster HPLC System (Hitachi High-Tech Science Co., Tokyo, Japan). HR-ESI-MS spectral data were collected using a Bruker micrOTOF QII (Bruker Co., Bremen, Germany) mass spectrometer. NMR spectra were recorded in acetone-*d_6_* using a Bruker AVANCE III 600 spectrometer. Optical rotations were measured using a JASCO P-2100 (JASCO Co., Tokyo, Japan) using a 10 mm length cell. UV spectra were measured using a JASCO V-550 UV-spectrometer (JASCO Co., Tokyo, Japan). Bioassay results were recorded on a Model 550 microplate reader (Bio-Rad, Hercules, CA, USA).

### 4.2. Marine Cyanobacterium M. producens

Samples of the marine cyanobacterium *M. producens* were collected from Kuba Beach, Nakagusuku, Okinawa, Japan in July of 2010. After freeze-drying, the samples were stored at −30 °C until the experiments were performed. Identification of the sample was accomplished via morphological observation under a microscope by Dr. Masayuki Fukuoka of Tokyo University of Marine Science and Technology. *Moorea producens* was a dominant cyanobacteria species in the sample. The sample also contained some unidentified diatoms.

### 4.3. Isolation of Oscillatoxin I

A frozen sample of the cyanobacterium *M. producens* (dry weight: 0.87 kg) was soaked for several days in ethanol at room temperature. After filtering the ethanol extract, it was extracted five times with methanol and once with acetone. The extracts were then combined and concentrated in vacuo to yield a residue (37.8 g), which was partitioned first between methanol/water (4:1, v/v) and hexane. The solvent of the 80% MeOH-soluble layer was then removed, and the remaining sample was partitioned using distilled water and ethyl acetate (EtOAc). The EtOAc layer was evaporated to dryness. The distilled-water layer was then dissolved with 1-butanol (BuOH) and separated into two extracts. Since the EtOAc layer of the extracts from the cyanobacterium *M. producens* showed the most potent bioactivity, this layer was separated using an open glass column (PEGASIL ODS, Senshu Co., Tokyo, Japan) measuring 20 × 120 mm with stepwise elution in 50%, 70%, 90%, and 100% methanol. The 70% methanol eluate was then purified via HPLC using a reversed-phase column (Cosmosil 5C18-AR-II, 10 × 250 mm, Nakalai Tesque Inc., Kyoto, Japan). Finally, oscillatoxin I (**1**, 1.1 mg) was isolated.

### 4.4. Biological Tests of Oscillatoxin I

Cytotoxicity assays against mouse L1210 leukemia cells were carried out for the isolated compounds. The growth inhibition activity of the compounds against the marine diatom *Nitzschia amabilis* were also evaluated. Both types of bioactive assays were performed using the XTT colorimetric reaction method as previously reported [30,31].

## Figures and Tables

**Table 1:** NMR data for oscillatoxin I in acetone-*d*_6_ (600 MHz for ^1^H and 150 MHz for ^13^C).

No.	δ_H_ Multiplicity (*J* in Hz)	δ_C_	No.	δ_H_ Multiplicity (*J* in Hz)	δ_C_
1	-	166.6, C	17	-	111.1, C
2	-	130.0, C	18	7.38 d (8.6)	133.4, CH
3	-	197.4, C	19	6.73 dd (3.1, 8.6)	116.3, CH
4	2.66 m	37.1, CH	20	-	157.6, C
5a	1.75 dd (13.7, 13.7)	45.3, CH_2_	21	6.97 d (3.0)	114.2, CH
5b	1.86 dd (4.8, 13.4)	-	22	0.94 d (6.6)	13.8, CH_3_
6	-	35.8, C	23	1.05 d (6.9)	17.4, CH_3_
7	-	164.0, C	24	1.21 s	28.3, CH_3_
8	6.19 dd (0.8, 16.0)	125.4, CH	25	1.31 s	24.7, CH_3_
9	6.07 dd (8.6, 16.0)	124.2, CH	26	1.08 d (6.6)	13.9, CH_3_
10	2.49 m	41.4, CH	27	-	174.1, C
11	3.25 dd (1.2, 11.3)	77.6, CH	28a	2.66 dd (1.1, 18.1)	36.3, CH_2_
12	1.61 m	24.7, CH	28b	3.07 dd (6.0, 18.1)	-
13a	1.59 m	35.4, CH_2_	29	5.54 m	72.4, CH
13b	1.59 m	-	30	4.84 m	78.7, CH
14a	1.67 m	34.1, CH_2_	31	1.43 d (6.6)	13.9, CH_3_
14b	1.67 m	-	32	3.22 s	56.4, CH_3_
15	4.47 dd (4.5, 7.5)	82.2, CH	11-OH	3.50 d (5.7)	-
16	-	143.0, C	20-OH	8.55 s	-

## Supplementary Materials

The following are available online at https://www.mdpi.com/2072-6651/11/6/366/s1, Figure S1: ESI-HRMS spectrum of oscillatoxin G in positive ion mode. Figure S2: ^1^H NMR spectrum of oscillatoxin I in acetone- *d_6_.* Figure S3: ^13^C NMR spectrum of oscillatoxin I in acetone- *d_6_*. Figure S4: ^1^H–^1^H COSY NMR spectrum of oscillatoxin I in acetone- *d_6_*. Figure S5: ^1^H–^13^C HSQC spectrum of oscillatoxin I in acetone- *d_6_*. Figure S6: ^1^H–^13^C HMBC spectrum of oscillatoxin I in acetone- *d_6_*.

## References

[1] Mynderse J.S., Moore R.E., Kashiwagi M., Norton T.R. (1977). Antileukemia activity in the Osillatoriaceae: isolation of debromoaplysiatoxin from *Lyngbya*. Science.

[2] Moore R.E., Blackman A.J., Cheuk C.E., Mynderse J.S., Matsumoto G.K., Clardy J., Woodard R.W., Craig J.C. (1984). Absolute stereochemistries of the aplysiatoxins and oscillatoxin A. J. Org. Chem..

[3] Cardellina J.H., Marner F.J., Moore R.E. (1979). Seaweed dermatitis: structure of lyngbyatoxin A. Science.

[4] Aimi N., Odaka H., Sakai S.I., Fujiki H., Suganuma M., Moore R.E., Patterson G.M.L. (1990). Lyngbyatoxins B and C, two new irritants from *Lyngbya majuscula*. J. Nat. Prod..

[5] Osborne N.J., Webb P.M., Shaw G.R. (2001). The toxins of *Lyngbya majuscula* and their human and ecological health effects. Environ. Int..

[6] Kimberly A.W., Marquart L., Norton S.A. (2012). Lyngbya dermatitis (toxic seaweed dermatitis) *Int*. J. Dermatol..

[7] Nagai H., Yasumoto T., Hokama Y. (1996). Aplysiatoxin and debromoaplysiatoxin as the causative agents of a red alga *Gracilaria coronopifolia* poisoning in Hawaii. Toxicon.

[8] Yasumoto T. (1998). Fish poisoning due to toxins of microalgal origins in the Pacific. Toxicon.

[9] Paerl H.W., Paul V.J. (2012). Climate change: links to global expansion of harmful cyanobacteria. Water Res..

[10] Nagai H., Watanabe M., Sato S., Kawaguchi M., Xiao Y.Y., Hayashi K., Watanabe R., Uchida H., Satake M. (2019). New aplysiatoxin derivatives from the Okinawan cyanobacterium *Moorea producens*. Tetrahedron.

[11] Fujiki H., Suganuma M., Nakayasu M., Hoshino H., Moore R.E., Sugimura T. (1982). The third class of new tumor promoters, polyacetates (debromoaplysiatoxin and aplysiatoxin), can differentiate biological actions relevant to tumor promoters. Gann.

[12] Fujiki H., Tanaka Y., Miyake R., Kikkawa U., Nishizuka Y., Sugimura T. (1984). Activation of calcium-activated, phospholipid-dependent protein kinase (protein kinase C) by new classes of tumor promoters: teleocidin and debromoaplysiatoxin. Biochem. Biophys. Res. Commun..

[13] Suganuma M., Fujiki H., Tahira T., Cheuk C., Moore R.E., Sugimura T. (1984). Estimation of tumor promoting activity and structure-function relationships of aplysiatoxins. Carcinogenesis.

[14] Arcoleo J.P., Weinstein I.B. (1985). Activation of protein kinase C by tumor promoting phorbol esters, teleocidin and aplysiatoxin in the absence of added calcium. Carcinogenesis.

[15] Nakamura H., Kishi Y., Pajares M.A., Rando R.R. (1989). Structural basis of protein kinase C activation by tumor promoters. Proc. Natl. Acad. Sci. USA.

[16] Pettit G.R., Herald C.L., Doubek D.L., Herald D.L., Arnold E., Clardy J. (1982). Isolation and structure of bryostatin 1. J. Am. Chem. Soc..

[17] Hennings H., Blumberg P.M., Pettit G.R., Herald C.L., Shores R., Yuspa S.H. (1987). Bryostatin 1, an activator of protein kinase C, inhibits tumor promotion by phorbol esters in SENCAR mouse skin. Carcinogenesis.

[18] Hale K.J., Hummersone M.G., Manaviazar S., Frigerio M. (2002). The chemistry and biology of the bryostatin antitumour macrolides. Nat. Prod. Rep..

[19] Basu A. (1993). The potential of protein kinase C as a target for anticancer treatment. Pharmacol. Therapeut..

[20] Antal C.E., Hudson A.M., Kang E., Zanca C., Wirth C., Stephenson N.L., Trotter E.W., Gallegos L.L., Miller C.J., Furnary F.B. (2015). Cancer-associated protein kinase C mutations reveal kinase’s role as tumor suppressor. Cell.

[21] Nakagawa Y., Yanagita R.C., Hamada N., Murakami A., Takahashi H., Saito N., Nagai H., Irie K. (2009). A simple analogue of tumor-promoting aplysiatoxin is an antineoplastic agent rather than a tumor promoter: development of a synthetically accessible protein kinase C activator with bryostatin-like activity. J. Am. Chem. Soc..

[22] Irie K., Yanagita R.C. (2014). Synthesis and biological activities of simplified analogs of the natural PKC ligands, bryostatin-1 and aplysiatoxin. Chem. Rec..

[23] Entzeroth M., Blackman A.J., Mynderse J.S., Moore R.E. (1985). Structures and stereochemistries of oscillatoxin B, 31-noroscillatoxin B, oscillatoxin D, and 30-methyloscillatoxin D. J. Org. Chem..

[24] Nagai H., Yasumoto T., Hokama Y. (1997). Manauealides, Some of the causative agents of a red alga *Gracilaria coronopifolia* poisoning in Hawaii. J. Nat. Prod..

[25] Chlipara G.E., Tri P.H., Hung N.V., Krunic A., Shim S.H., Soejarto D.D., Orjala J. (2010). Nhatrangins A and B, aplysiatoxin-related metabolites from the marine cyanobacterium *Lyngbya majuscula* from Vietnam. J. Nat. Prod..

[26] Gupta D.K., Kaur P., Leong S.T., Tan L.T., Prinsep M.R., Chu J.J.H. (2014). Anti-Chikungunya viral activities of aplysiatoxin-related compounds from the marine cyanobacterium *Trichodesmium erythraeum*. Mar. Drugs.

[27] Han B.N., Liang T.T., Keen L.J., Fan T.T., Zhang X.D., Xu L., Zhao Q., Wang S.P., Lin H.W. (2018). Two marine cyanobacterial aplysiatoxin polyketides, neo-debromoaplysiatoxin A and B, with K^+^ channel inhibition activity. Org. Lett..

[28] Tang Y.H., Liang T.T., Fan T.T., Keen L.J., Zhang X.D., Xu L., Zhao Q., Zeng R., Han B.N. (2019). Neo-debromoaplysiatoxin C, with new structural rearrangement, derived from debromoaplysiatoxin. Nat. Prod. Res..

[29] Tang Y.H., Wu J., Fan T.T., Zhang H.H., Gong X.X., Cao Z.Y., Zhang J., Lin H.W., Han B.N. (2019). Chemical and biological study of aplysiatoxin derivatives showing inhibition of potassium channel Kv1.5. RSC Adv..

[30] Kawabata T., Lindsay D.J., Kitamura M., Konishi S., Nishikawa J., Nishida S., Kamio M., Nagai H. (2013). Evaluation of the bioactivities of water-soluble extracts from twelve deep-sea jellyfish species. Fish. Sci..

[31] Jiang W., Akagi T., Suzuki H., Takimoto A., Nagai H. (2016). A new diatom growth inhibition assay using the XTT colorimetric method. Comp. Biochem. Physiol. Part C.

