# Supplementary material for: Oscillatoxin I: A New Aplysiatoxin Derivative, from a Marine Cyanobacterium
Source: Toxins (Basel). 2019 Jun 21;11(6):366. doi: 10.3390/toxins11060366 (PMC6628398; doi:10.3390/toxins11060366)
Supplement: Supplementary file 1 [file toxins-11-00366-s001.pdf]

Supplementary Materials

Oscillatoxin I, a New Aplysiatoxin Derivative, from a Marine Cyanobacterium

Hiroshi Nagai, Shingo Sato, Kaori Iida, Kazutaka Hayashi, Mioko Kawaguchi, Hajime Uchida and Masayuki Satake

Figure S1. ^{1}H NMR spectrum of oscillatoxin I in acetone- d_6.
Figure S2. ESI-HRMS spectrum of oscillatoxin I in positive ion mode.
Figure S3. ^{1}H–^{13}C HSQC spectrum of oscillatoxin I in acetone- d_6.
Figure S4. ^{1}H–^{13}C HMBC spectrum of oscillatoxin I in acetone- d_6.
Figure S5. ^{1}H–^{1}H COSY NMR spectrum of oscillatoxin I in acetone- d_6.
Figure S6. ^{13}C NMR spectrum of oscillatoxin I in acetone- d_6.

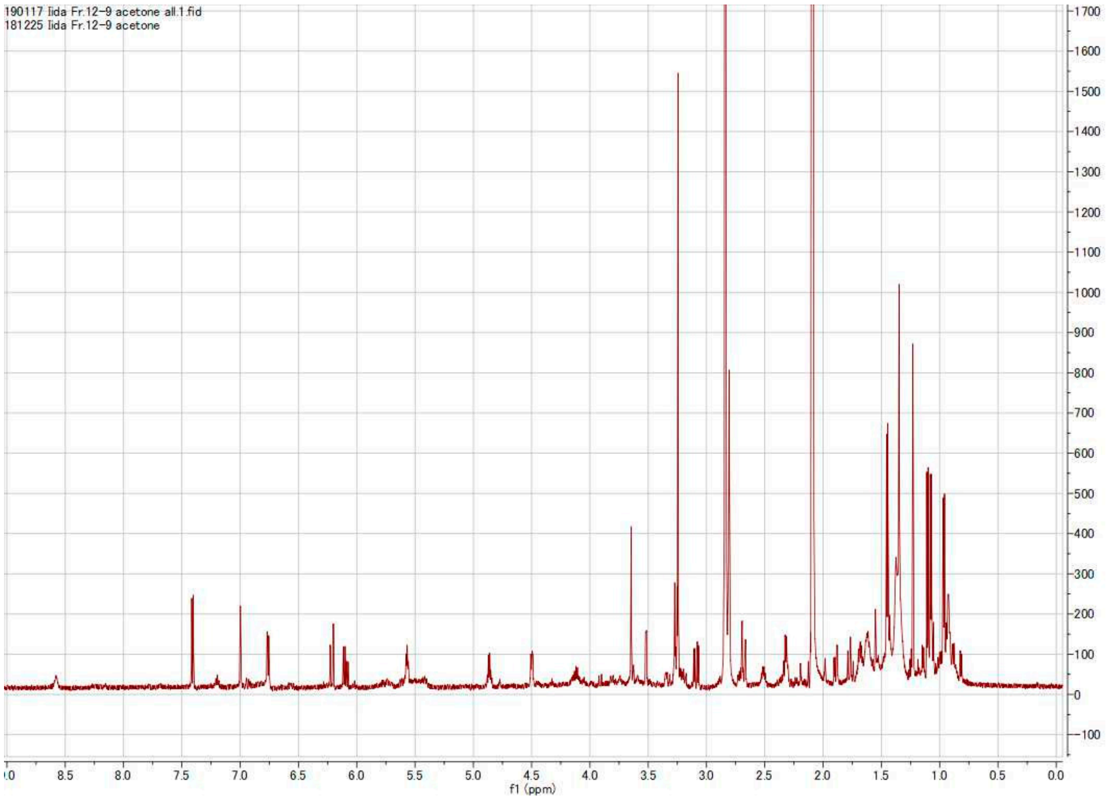

Figure S1. ^{1}H NMR spectrum of oscillatoxin I (**1**) in acetone-d_6.

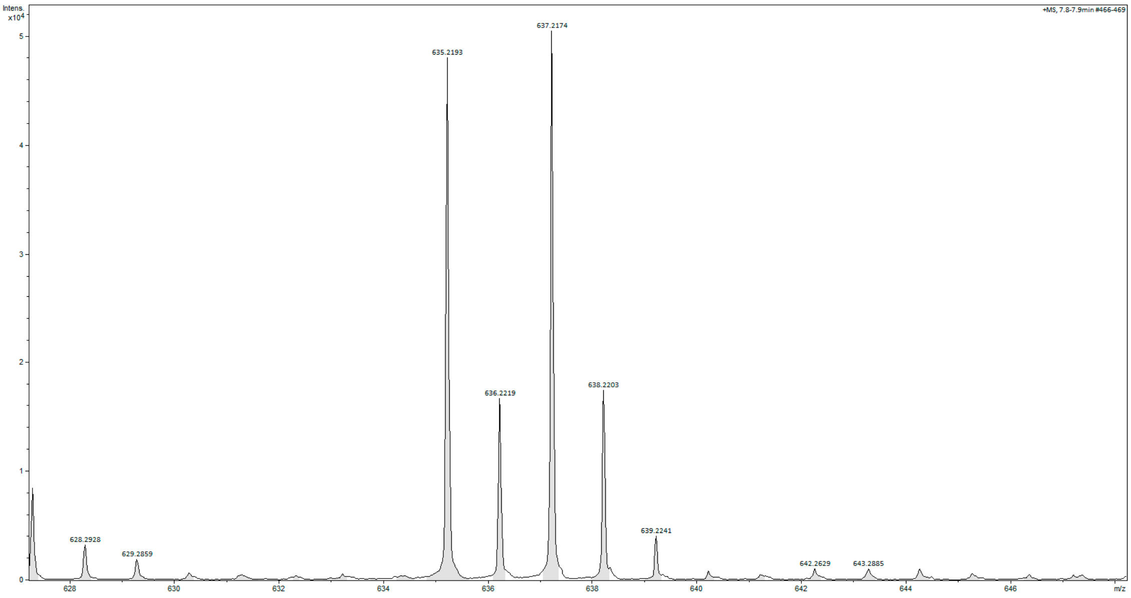

Figure S2. ESI-HRMS spectrum of oscillatoxin I (**1**) in positive ion mode.

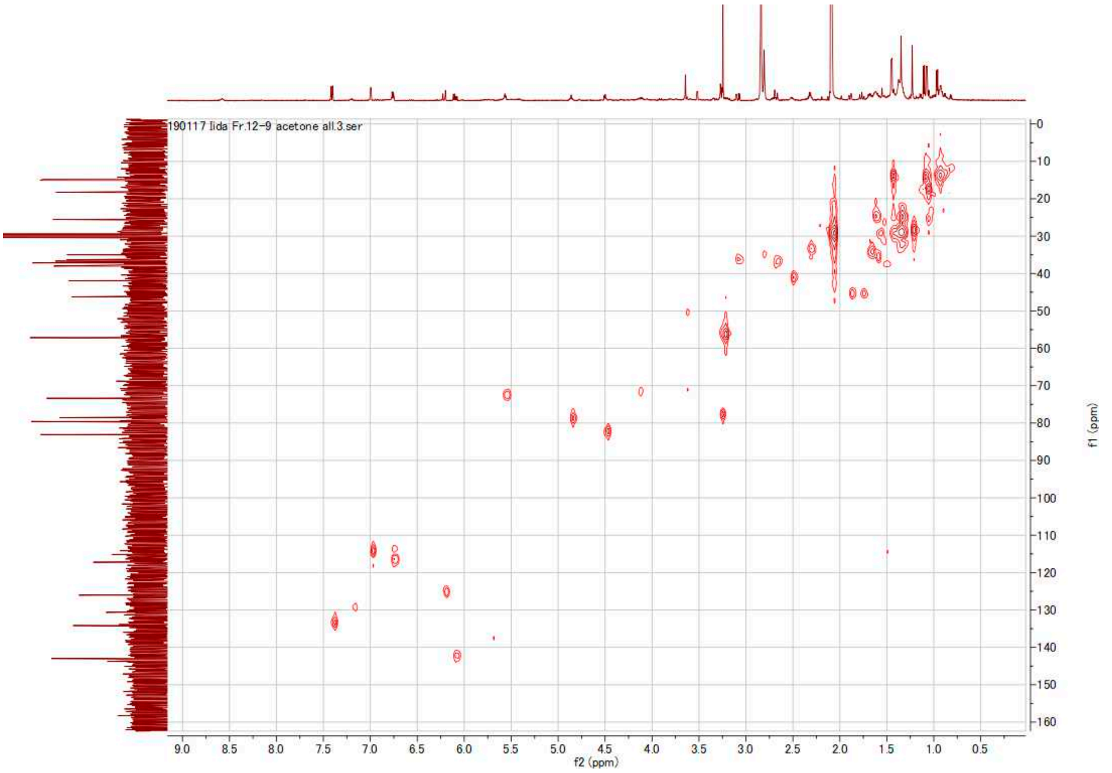

Figure S3. ^{1}H–^{13}C HSQC spectrum of oscillatoxin I (**1**) in acetone- *d*6.

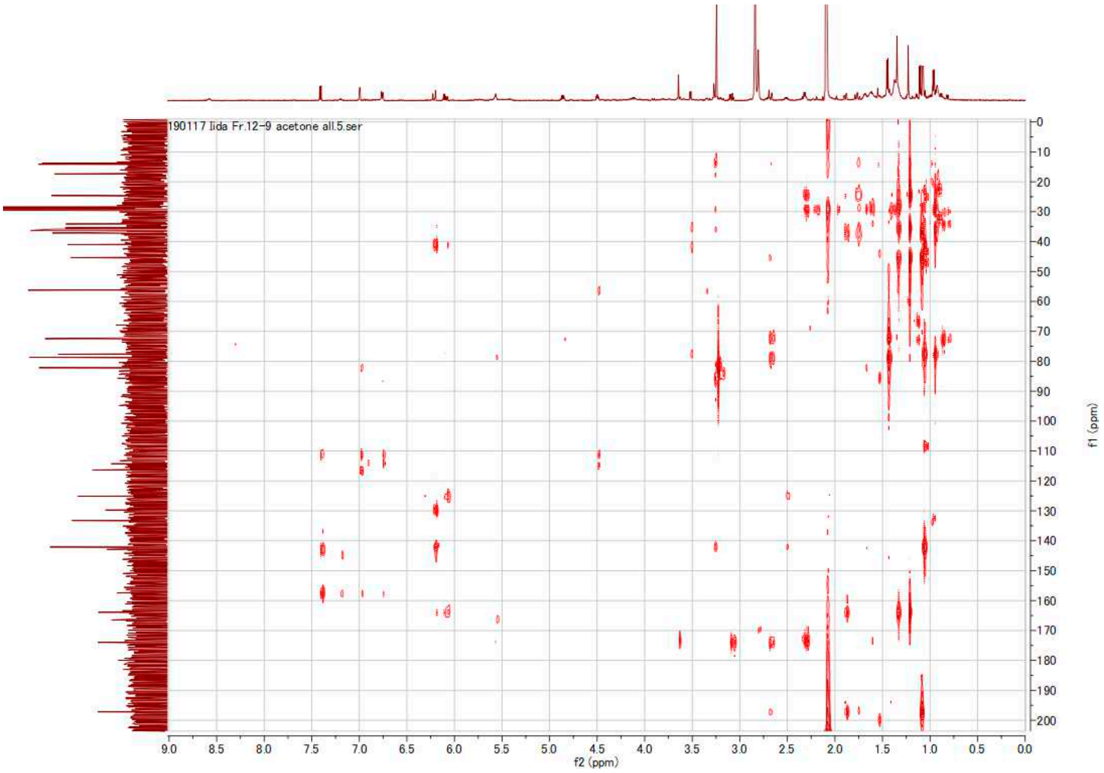

Figure S4. ^{1}H–^{13}C HMBC spectrum of oscillatoxin I (**1**) in acetone- *d*6.

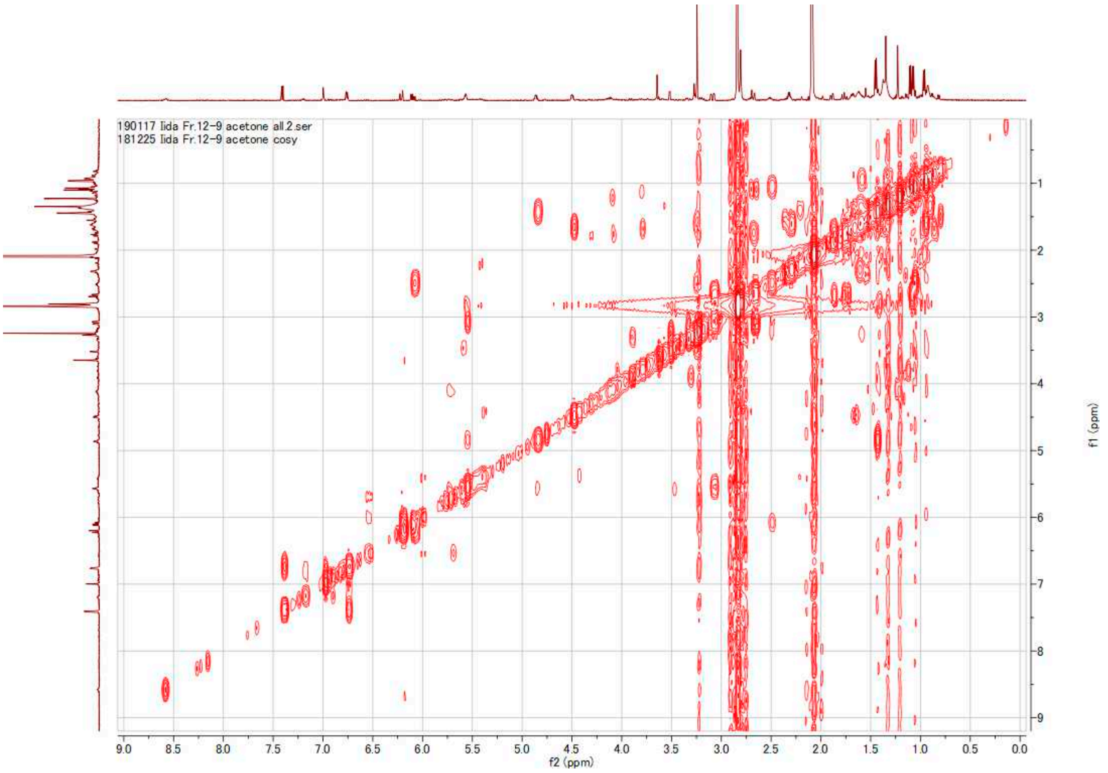

Figure S5. ^{1}H–^{1}H COSY NMR spectrum of oscillatoxin I (**1**) in acetone- *d*6.

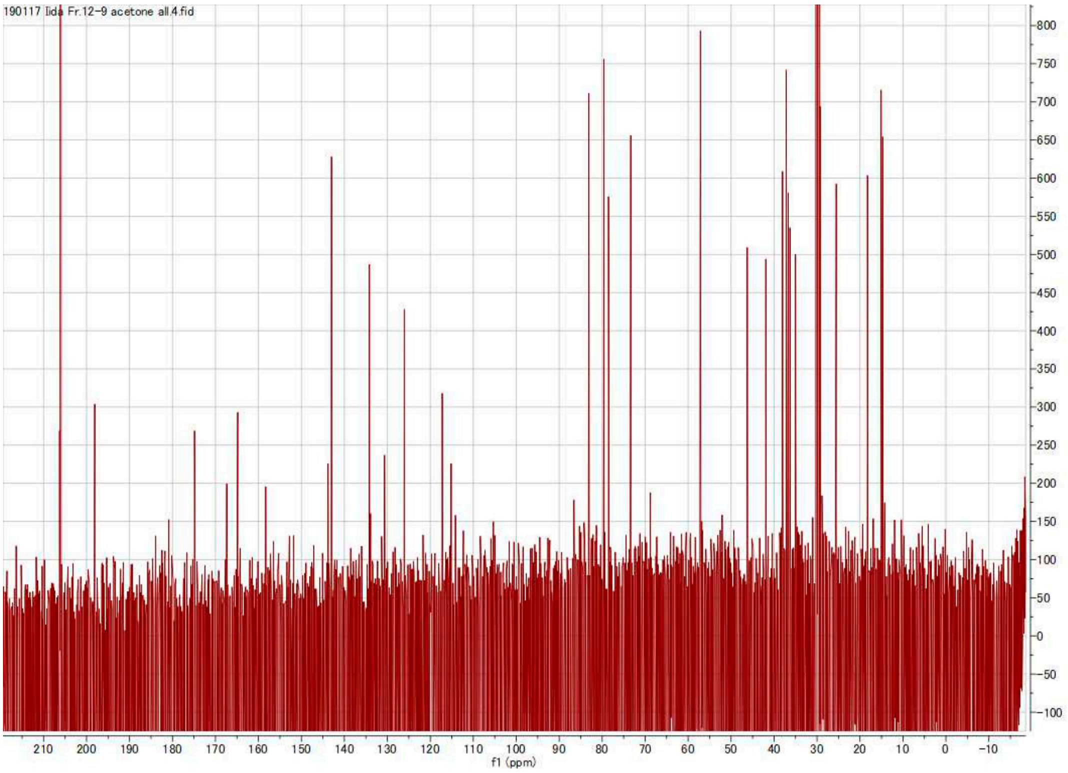

Figure S6. ^{13}C NMR spectrum of oscillatoxin I (**1**) in acetone- *d*6.
